# Staged Radiosurgical Ablation for Choroid Melanoma: A Case Report with Emphasis on the Role of Patient Preparation, Treatment Planning, and Precision of Delivery

**DOI:** 10.7759/cureus.611

**Published:** 2016-05-16

**Authors:** Marta Adamczyk, Piotr Janiga

**Affiliations:** 1 Medical Physics Department, Greater Poland Cancer Centre; 2 Radiotherapy Ward I, Greater Poland Cancer Centre

**Keywords:** cyberknife, orbital tumors, fixation devices

## Abstract

The aim of reporting this case of choroid melanoma of the left eye is to introduce the in-house-designed treatment planning protocol for fractionated radiosurgical ablation of an intraocular lesion. This is a clinical case with emphasis on treatment preparation and delivery using the Accuray CyberKnife Robotic Radiosurgery System (Accuray, Sunnyvale, CA, USA) for a patient immobilized with a head mask and our in-house-made eye fixation system.

## Introduction

The Accuray CyberKnife Robotic Radiosurgery System uses several hundred treatment beams selected during inverse treatment planning from more than one thousand possible beam directions. The linear accelerator attached to the flexible robotic arm enables the irradiation of the patient in a non-isocentric mode using different sizes of circular collimators without intensity modulation. Due to these technological features, theoretically, it is possible to deliver a highly conformal, uniform dose with step dose gradients to all stereotactic targets throughout the patient's body [1]. It raises the question whether this system can be used to irradiate the irregularly shaped lesions located in the eye. The answer should take into account the fact that the potentiality of the CyberKnife treatment makes it very important to provide a proper immobilization and position verification of the target throughout the whole irradiation period. Additionally, the treatment of intraocular tumors is more complicated due to the effects of eyeball movement [2]. Thus, treatment with external beam radiation therapy in such a location brings about a serious concern of inducing optic neuropathy, retinopathy or dry eye syndrome [3]. Without additional fixation systems, often based on in-house-made equipment and implementation procedures, the CyberKnife’s high degree of dose conformity and the best dose distribution cannot be realized. Thus, the aim of this case report is to introduce the in-house-designed treatment planning protocol for fractionated radiosurgical ablation of an intraocular lesion. We present a clinical case with emphasis on treatment preparation and treatment delivery with a CyberKnife Robotic Radiosurgery System. Informed consent was obtained from the patient for this study.

## Case presentation

**Figure 1:**
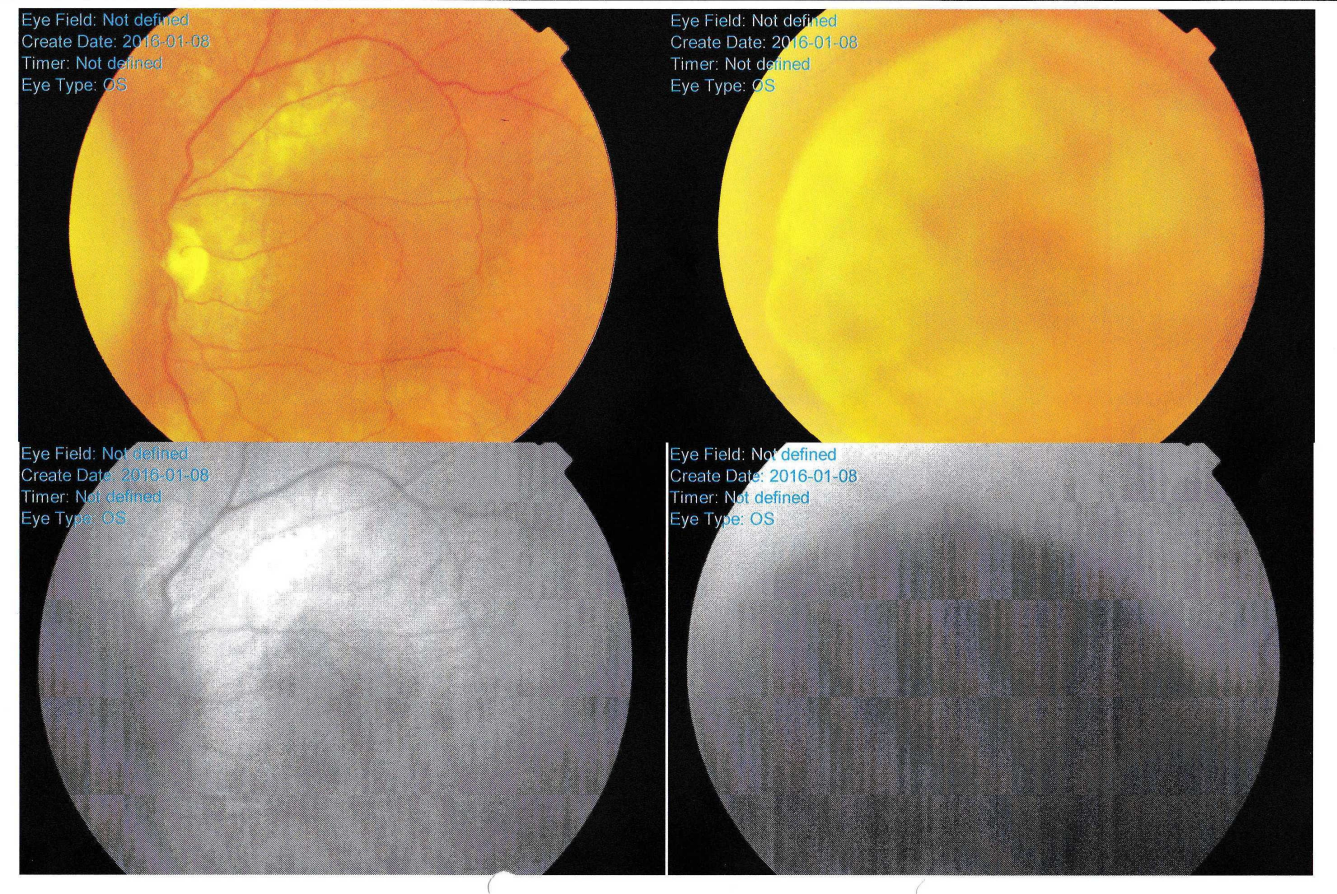
Ophthalmoscopic View of a Left Eye Fundus (Fundoscopy) Field of view was small due to magnification. The border of the left tumor is seen in the first photo and the whole melanoma is clearly visible in the second one. Bottom photos were taken in infrared.

**Figure 2:**
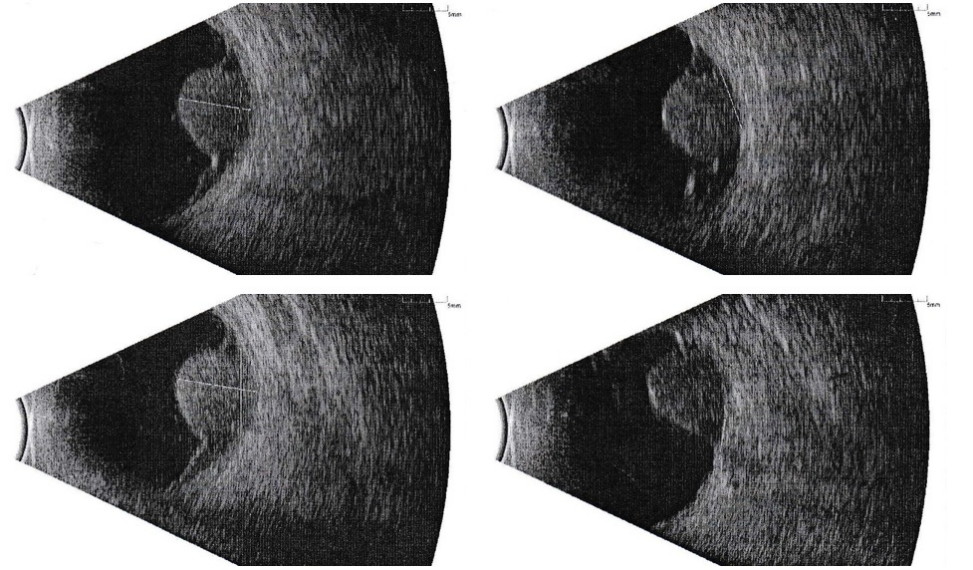
Ultrasound of Left Eyeball Tumor width and thickness were taken from this examination.

A 57‑year‑old male was referred to our center with a diagnosis of choroid melanoma of the left eye. Detailed ophthalmologic data was provided by the primary clinic. It included the tumor’s width, length, thickness, position, and relations to the optic disc and macula. A circular-shaped tumor was located just right of the optic disc (Figure 1). The lesion was measured during ultrasound examination (Figure 2), revealing retinal detachment as well.

Due to the retinal detachment, the visual field was limited to medial quadrants. Extraocular extension as well as metastasis were excluded.

The patient was qualified to receive a staged radiosurgical ablation for choroid melanoma of the left eye. In this case, plaque brachytherapy seemed to be suboptimal, due to tumor thickness and proximity to the optic nerve. A reasonable alternative treatment could be proton therapy, however, it is hardly available.

**Figure 3:**
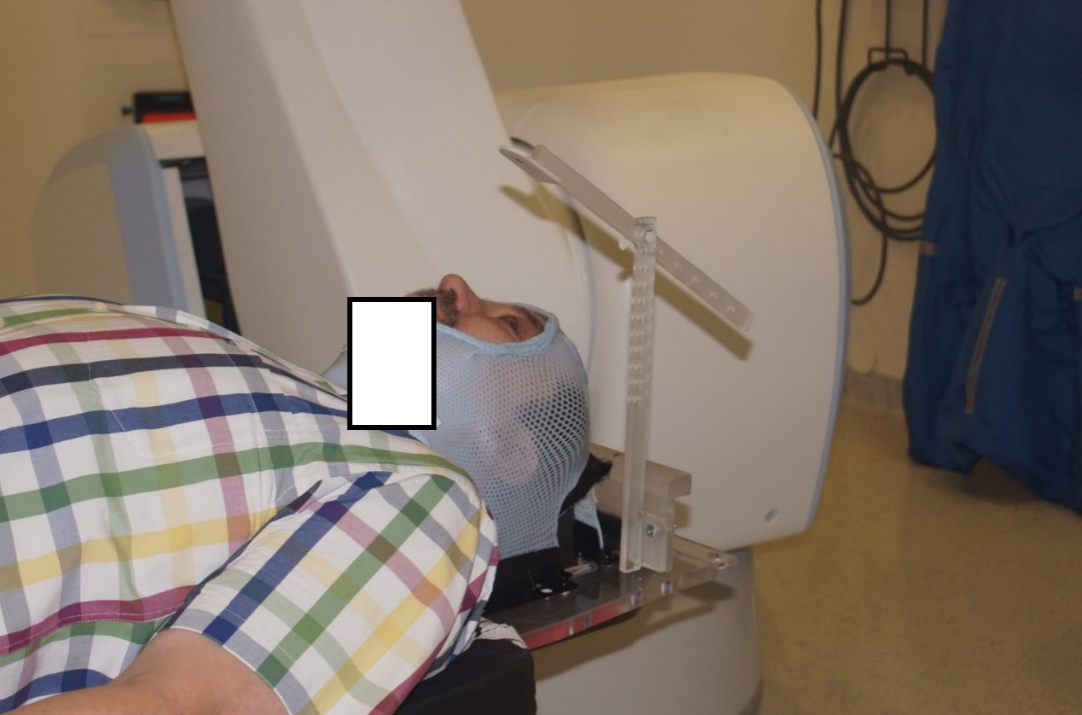
Patient Immobilized with a Head Mask and our In-house-made Eye Fixation System

The first step in treatment preparation was the making of a head mask. The patient was positioned precisely in a standard supine position and a thermoplastic mask was molded to the head with a hole for both eyes, nose, and mouth. After being thus immobilized, the patient was instructed to fix his sight on one designated point in the in-house eye fixation system. This immobilization device, fully designed and constructed at our hospital, was made with polymethyl methacrylate (PMMA). The main portion of the device consists of a stabilizing element, as it enables to attach the immobilization device to the treatment couch. It is a base for the second element, the vertical bar. In the upper part of the vertical bar, the second bar (a horizontal one) is fixed. A black spot is placed on the second end of the horizontal bar, on a bright PMMA piece. That is the element/spot that holds the patient's sight finally fixating both lens positions. The immobilization device is always placed above the eye in which the tumor is located (Figure 3).

Consequently, for all patients who have vision in both eyes (like in the presented case), the lens located in the healthy eye would be directed inwards.

The thin-sliced (with 1 mm slice spacing) high-resolution planning computed tomography (CT) images were acquired on Somatom Emotion Duo (Siemens, Erlangen, Germany). Precise scans were obtained through the whole head and neck up to the shoulder line, for the patient lying in a treatment position with the eye fixation system.

Before planning CT acquisition, as well as before each treatment fraction, the patient was instructed to focus his sight by looking at the designated point on the eye fixation device. To improve the patient’s comfort during the CT scanning and irradiation process, a knee support was used.

Target volumes and critical structures were delineated manually by the radiation oncologist on axial images with simultaneous overlay of the outlines on coronal and sagittal reconstructions [3]. For the presented clinical case, there were no problems with viewing details of the target, optic nerves, and chiasm. Hence, diagnostic magnetic resonance imaging (MRI) was not performed. Treatment volumes were created first by outlining the gross tumor volume (GTV), which consisted of intraocular tumor. To improve tumor delineation, detailed ophthalmologic data was used. Choroid melanomas are visible in details in ocular examination and in ultrasound. These combined modalities are more valuable than CT or MRI and form the basis for preparing tumor contours. Then, GTV was expanded 2-mm isotropically to create the planning target volume (PTV).

The organs at risk (OARs) outlined for this clinical case included the left eye, right eye, left lens, right lens, both optic nerves and their chiasm, left lacrimal gland, and brainstem. For those structures, a series of dose constraints were specified, to ensure adequate target irradiation and a minimal dose to critical structures. A cumulative dose of 50 Gy was precisely prescribed to 80% isodose and delivered in five fractions, giving 10 Gy per fraction. The dose delivered to OARs was minimized to fulfill constraints presented in Table 1. Dose constraints were adopted to our hospital protocol according to the guidelines proposed by Grimm et al. [4].

**Table 1:** Dose Constraints from our Hospital Protocol for the Treatment of an Intraocular Lesion in 5 Fractions V_YcGy_- volume receiving at least Y cGy; D_max_ - the maximum dose received by the analyzed organ.

Organ at risk	Constraints
Optic chiasm	D_max_<2500cGy
	V_2000cGy_<0.2cc
Optic nerve	D_max_<2600cGy
	V_2500cGy_<0.03cc
	V_2000cGy_<0.05cc
	V_1250cGy_<0.5cc
Lacrimal gland	D_max_<1500cGy
Brainstem	D_max_<3100cGy
Lenses	D_max_<700cGy

Additionally, after fulfilling the above-mentioned constraints, we also checked how a big dose is delivered to presented volumes of each OAR.

As the tumor was located in the eye (proximity to the left optic nerve), an individual therapeutic decision was taken. The aim was to spare the portion of the left optic nerve opposite to the tumor. This part of the nerve conducts neuronal stimuli from the left half of a retina, not invaded by melanoma. Thus, some useful vision in the nasal quadrants of the treated eye can still can rescued, minimizing a dose delivered to the left half on the nerve (precise values are specified in Table 1). Secondly, emphasis was put on achieving the PTV coverage higher than 90% with the left lens spared as much as possible. With all those criteria, the treatment plan was calculated using the MultiPlan 4.6.0 Treatment Planning System (TPS) (Accuray, Sunnyvale, USA) based on a head single path with 6D Skull Tracking algorithm.

**Figure 4:**
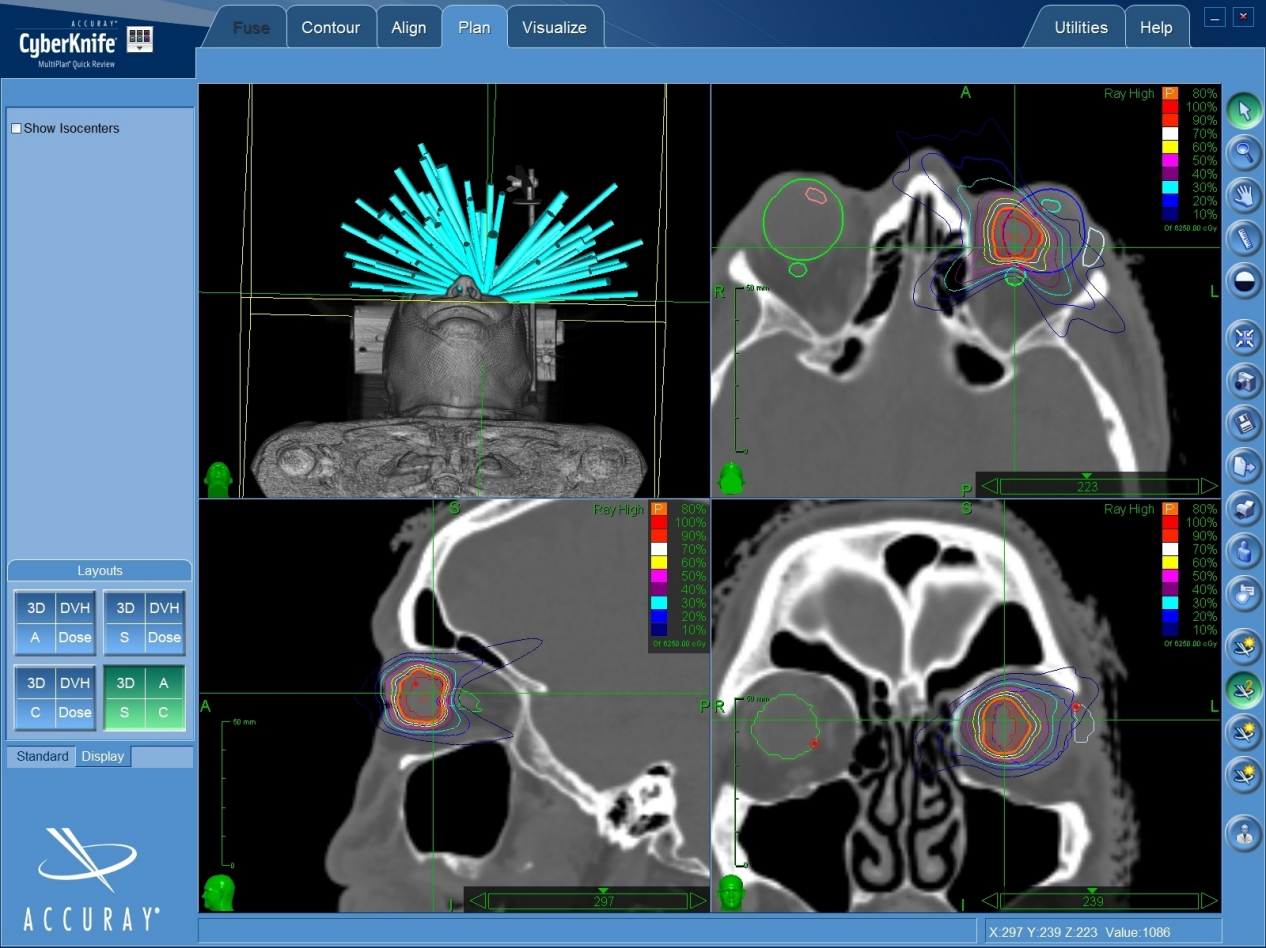
The Beams' Entrances and Isodose Distributions in the Axial, Sagittal, and Coronal Planes Through the Tumor Volume

Out of 12 collimator sizes (with diameters ranging from 5 mm to 60 mm) available with the CyberKnife radiosurgical system, for the 2.25‑cc PTV, IRIS collimators with diameters of 7.5 mm, 10.0 mm, 12.5 mm, and 15.0 mm were used. The chosen collimator sizes were smaller than or approximately equal to the maximum length of the prescribed target (PTV) [1]. The performed planning was conventional in terms of preparing a non-isocentric treatment. An inverse planning method was used to choose the beams delivering the dose by 6-MV photons [1]. Finally, the treatment plan required 79 beams, coming from 43 nodes, with the estimated treatment time per fraction of 26 minutes. This time reported in MultiPlan TPS included the five minutes provided for patient set-up and was calculated for the image time interval of 60 seconds. Dose volume histogram (DVH) parameters and visual inspection of isodose distribution were evaluated for the acceptance of the prepared treatment plan. The beams' entrances and isodose distributions in the axial, sagittal, and coronal planes through the tumor volume are presented in Figure 4.

All the doses fulfilled tolerance levels, except the dose to the left lens. One of the most critical conditions, namely the dose delivered to the left optic nerve, was fulfilled by giving this nerve the maximum dose of 2524.03 cGy, minimizing the dose received by specified volumes. The dose delivered to all other evaluated OARs did not reach the maximum permissible dose values. Detailed information about descriptive statistics for DVH parameters are presented in Table 2.

**Table 2:** DVH Parameters According to Prepared Treatment Plan V_YcGy_- volume receiving at least Y cGy, D_max_ - the maximum dose received by the analyzed organ), D_Zcc_ - the dose received by Z cc of the analyzed organ.

Organ at risk	DVH value
Optic chiasm	D_max_=183.01cGy
	D_0.2cc_=131.00cGy
Left optic nerve	D_max_=2524.03cGy
	V_2500cGy_=0.005cc
	V_2000cGy_=0.024cc
	D_0.03cc_=1805.40cGy
	D_0.05cc_=1455.40cGy
	D_0.5cc_=422.40cGy
Right optic nerve	D_max_=368.03cGy
	D_0.03cc_=227.50cGy
	D_0.05cc_=186.90cGy
	D_0.5cc_=52.80cGy
Lacrimal gland	D_max_=1055.54cGy
Brainstem	D_max_=147.63cGy
Left lens	D_max_=747.98cGy
Right lens	D_max_=5.82cGy

Focusing on doses delivered to targets, the minimum dose equaled 5012.78 cGy and 3147.84 cGy for GTV and PTV, respectively, giving a 100.00% coverage for GTV and 92.74% coverage for PTV. When the nerve was excluded from the PTV contour with the margin of 2, 3, and 4 mm, the coverage value increased to 94.22%, 96.02%, and 96.48%, respectively. Such exclusion decreased the volume of PTV from 2.25 cc (for the whole PTV), through 2.21 cc (for PTV minus the nerve with a 2-mm margin) and 2.16 cc (for PTV minus the nerve with a 3-mm margin) to 2.09 cc (for PTV minus the nerve with a 4-mm margin). The conformity index calculated for the whole PTV (without any exclusions from its volume) equaled 1.15, whereas its homogeneity index was reported to be 1.25.

6D Skull Tracking algorithm was used to track and correct the position of the visualized radiographic landmark of the cranium based on the imaging performed by a pair of orthogonal diagnostic X-ray tubes and corresponding image detectors [5]. Literature data for phantom studies showed that the precision of 6D Skull Tracking is comparable to frame-based systems thus eliminating the necessity for using invasive headframes [1]. The geometric verification was performed several times per minute during the whole radiotherapy fraction. An eye fixation system was used independent of the skull position verification. Positioning details (time, number of acquired images) as well as final treatment delivery with the number of acquired images are presented in Table 3.

**Table 3:** The Time and Number of Verification Images Acquired During Patient Positioning and Irradiation

Fraction no	Positioning		Treatment	
	Time [min]	No of images	Time [mm:ss]	No of images
1	2	8	19:59	44
2	2	6	19:08	32
3	2	8	19:03	32
4	3	6	19:14	30
5	3	10	18:50	30

Irradiation was performed twice a week: on Tuesday and Thursday. Before each fraction, the patient was instructed to fix the sight on the specified element (black spot) of our eye fixation system. He was also informed that he might experience eyestrain, and if so, the treatment would be interrupted and continued after a short break. The patient tolerated the treatment well and reported no complaints (no break was needed).

## Discussion

CyberKnife treatment represents a favorable therapeutic option for different types of intraocular tumors as it offers a dose distribution characterized by high degree of conformity and protection of organs at risk [6]. This makes it a valuable alternative to surgery (eye enucleation) or other radiotherapy techniques (brachytherapy, proton therapy) [7].

In response to all issues related to in-treatment patient position verification, an eye fixation system was used independent of skull positioning. This system was not placed at extreme angles (as it is often used in proton therapy), but approximately at the level of the pupil in a normal eyeball position, which was extremely important for the patient’s comfort [8]. Moreover, the system does not contain any light for the purpose of eye fixation. It reduces the patient’s strain due to concentration on a selected element in a required time period [8]. The competitive fixation option was proposed by the group from Munich. They used a frameless robotic radiosurgery system with retrobulbar anaesthesia to treat their patient [9]. After such an injection, a complete akinesia of the globe within the orbit is achieved and this transition state is used to perform the MRI, CT planning imaging, and irradiation [9-10]. On the one hand, such positioning represents a very interesting fixation possibility, but on the other hand, it is a challenge to introduce this system without an ophthalmology department nearby.

Another challenging usable option worth mentioning is irradiation combined with hyperthermia, reported for the orbital metastasis of breast carcinoma [11]. Pre-treatment hyperthermia seems to be very promising but its restriction by the tumor location (precisely, its depth from the skin) and the avoidance of microwave-induced high temperatures reaching the lens, makes it an option limited to selected patients.

With the use of an eye fixation system during the CT acquisition, there was no physical interference with the gantry. After a CT reconstruction, no artifacts of an eye fixation system were recorded. To make sure that dose distribution was not disturbed by the fixation device, the whole system was contoured and blocked for the beams going through it. It is a commonly reported practice by which intersection with eye fixation system elements is avoided; however, absorption of the 6 MV photon beams is negligible [8].

Technically, a fixation device was attached to the treatment table in the same way as a mask. A fixation element (black spot) was located above the treated eye within a distance that was indicated by the patient as a comfortable one. Consequently, the unaffected eye (precisely lens) was also directed towards the eye fixation element. This can be regarded as risky because in such a positioning the unaffected eye could be exposed to higher dose than normally received when looking straight ahead. Therefore care was taken to exclude the unaffected eye from the physical path of the beams by blocking the beams that could pass through it. Consequently, the right eye and the right lens of the patient received the maximum dose of 12.21 cGy and 5.82 cGy, respectively.

Unfortunately, for a tumor of this size, it was not possible to obtain such a low dose in the lens of the diseased eye. Moreover, the dose for the lens was increased to the maximum dose of 747.98 cGy (47.98 cGy exceeding the tolerance dose defined in the protocol) to achieve the PTV coverage higher than 90% (precisely, it was 92.74%).

In the presented fixation system, a patient has an active role to play during the whole therapy. As a result of the training (before CT acquisition he focused his sight on a black spot on the fixation system), the patient indicated the comfortable distance between the eye and the central element of a fixation system. Then, he was told that he could interrupt a treatment by raising his hand during each fraction.

The other aspect that should be discussed is relevant to patients with claustrophobia. To give the patient more space, a hole in a mask was made for both eyes, nose, and mouth. Originally, we prepared a mask with a hole for diseased eye and nose, but then an opening was enlarged to the unaffected eye and mouth to improve the patient’s comfort. It has no impact on mask stability as its material still immobilized the left and right side of the head as well as its apex. In the bottom part of the head, the mandible was also well stabilized.

Finally, single treatment fraction time took not more than 20 minutes with the additional 2-3 minutes needed for positioning. The patient had not recorded any complaints related to treatment procedure. We observed that the CyberKnife dose delivery system offers advantages in a treatment of intraocular lesions and our preliminary results appear to be very promising.

## Conclusions

The analysis presented a possible way of treating patients with intraocular lesions. Such treatment, prepared and verified according to our protocol with CyberKnife Robotic Radiosurgery System, and the use of an in-house-made eye fixation system has a potential to deliver accepted doses to the PTV while at the same controlling a dose delivered to critical structures located in this region. Due to the eye fixation method used, such aggressive radiotherapy with large fraction size is dedicated to cooperating patients, as the patient’s role during the treatment preparation and realization is very active.
